# Wafer-scale integration of photonic integrated circuits and atomic vapor cells

**DOI:** 10.1515/nanoph-2025-0500

**Published:** 2025-12-05

**Authors:** Arieh Grosman, Roy Zektzer, Noa Mazurski, Liron Stern, Uriel Levy

**Affiliations:** 26742 Institute of Applied Physics, The Faculty of Science, The Center for Nanoscience and Nanotechnology, The Hebrew University of Jerusalem, Jerusalem, Israel; Faculty of Engineering, Bar-Ilan University, Ramat-Gan 5290002, Israel

**Keywords:** nanophotonics, wafer scale, atomic vapor

## Abstract

Atom-based technologies have played a central role in both fundamental research and application-driven developments. For example, devices such as atomic clocks and magnetometers are essential for precision time-keeping, navigation, and sensing. However, many of these demonstrations remain confined to laboratory settings due to their reliance on bulky equipment and centimeter-scale atomic vapor cells. In recent years, significant efforts have been made to miniaturize these vapor cells to enable field-deployable systems. Yet, integrating these cells with the necessary photonic components remains a complex and non-scalable process. To address this challenge, we have introduced the atomic-cladded waveguide (ACWG) architecture, which enables the integration of atomic and photonic functions on the same chip. While the ACWG concept provides a significant step forward toward integration, there is still a significant gap related to wafer scale manufacturability. In particular, previous demonstrations of atomic–photonic integration have relied on manual assembly of vapor cells onto single chips, restricting miniaturization, manufacturability, and thermal robustness. To revolutionize manufacturability of these devices, we hereby demonstrate our new generation of ACWG devices that overcomes these constraints. The approach is based on wafer bonding of a silicon wafer – consisting of multiple photonic chips to a glass wafer with pre-etched atomic chambers. This wafer-scale process yields multiple miniaturized integrated photonic–atomic chips in a single batch. The bonded devices operate reliably at elevated temperatures over an extended period of time, allowing higher atomic densities to be used. The fabrication method consists of well-defined, repeatable steps, paving the way for scalable production of mature integrated photonic–atomic systems for next-generation sensing, metrology, and quantum technologies, inspired by commercial complementary metal-oxide-semiconductor-based processes.

## 1 Introduction

**Figure 1:**
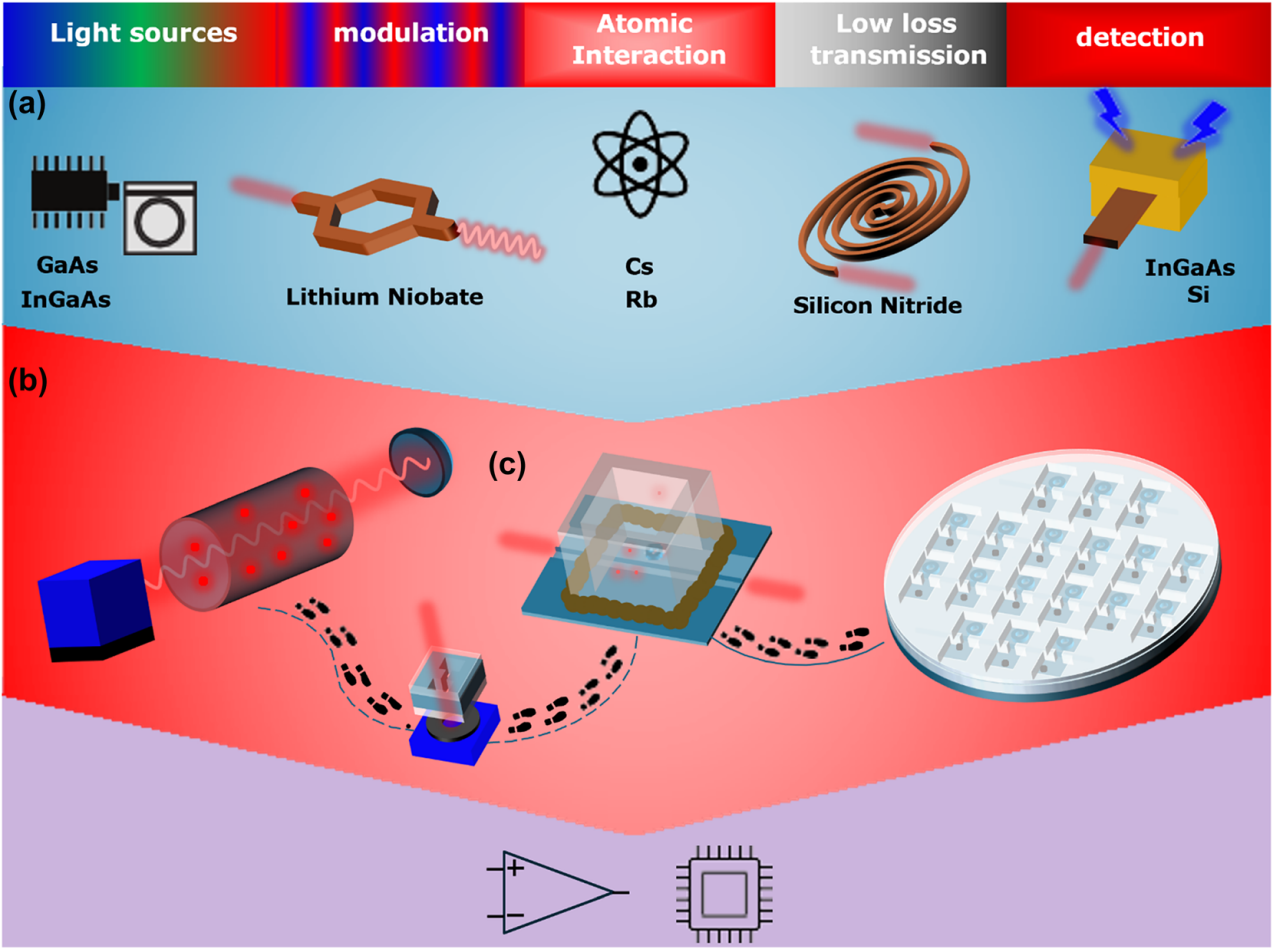
The evolution of vapor cells. (a) Atomic interaction on a photonic chip requires the integration of various building blocks – such as lasers, modulators, waveguides, and detectors – often fabricated from different materials. (b) Schematic of a traditional centimeter-scale atomic measurement system, featuring a modulated light source directed through a Rb vapor cell and onto a detector. (c) Road map toward miniaturization of Rb cell photonic integration starting from millimeter-sized bonded cells, progresses to glued photonic atomic cladding waveguides, to finally achieving a wafer-scale photonic–atomic platform.

Alkali vapors interaction with light have been studied over the last several decades due to their multiple applications in metrology and quantum physics, with applications such as atomic clocks [1], magnetometers [2], and RF sensors [3] to name a few. The interaction of light with rubidium (Rb) alkali vapor can serve as a basic building block for atomic clocks [1] that are used as precise frequency and time references in numerous applications such as telecommunications, network synchronization, satellite navigation, and more. Most atomic-based devices such as sensors and clocks require a light source, light modulation, being either frequency, phase, or amplitude modulation, interaction section between light and vapor, and light detection (Figure 1(a)). In order to make quantum sensors an accessible technology, these sensors should be fabricated in large numbers and at low cost. Ideally, all the required optical elements, lasers, modulators, atomic interaction, and photodetection, should be fabricated in the same process and integrated over the same platform. Over the last decade, significant advancements have been made in light manipulation at the chip scale. In fact, the emerging field of silicon photonics is evolving into an enabling technology in creating and successfully exhibiting a range of various nanoscale devices such as lasers [4], modulators [5], and photodetectors [6]. These days, several entities are offering multi-project wafer service (MPW) with several optical materials such as silicon nitride (SIN) for low-loss light propagation [7], lithium niobite for phase modulation [8], and III–V material bonding to silicon for integrated photodetectors and lasers [9]. Over the past decade, significant progress has been made in the miniaturization of vapor cells, evolving from traditional glass-blown (Figure 1(b)), centimeter-scale devices to the microfabrication of microelectromechanical systems-based structures [10], [11], [12] and atomic cladded waveguides (ACWGs) [13]. This progress has enabled reduced power consumption, increased portability, and greater versatility in end-user applications (Figure 1(c)).

The ACWGs [13], [14] have shown interesting features and already have been used for applications such as strong coupling, extreme nonlinearity [15], [16], optical isolation [17], frequency referencing [18], [19], and quantum optics [20], demonstrating its potential to be a major building block for an atomic–photonic chip system. However, wafer-scale fabrication of these devices remains a challenge in the transition from laboratory experiments to a viable product. While researchers have successfully tackled the challenge of manufacturability and miniaturization for microfabrication vapor cells at the millimeter-scale [21], [22], [23], combining wafer-scale miniaturized vapor cells with silicon complementary metal-oxide-semiconductor (CMOS)-compatible photonic integrated circuits remains a challenge. Mitigating this challenge is expect to enable a new type of devices supporting diverse different applications such as chip-based lasers [24], lithium niobate electro-optic modulators [25], stabilized atomic spectroscopy [26], and photodetectors [6] on low-loss waveguides platform [27], directly connected to standard silicon readout integrated circuit (ROIC).

Motivated by the acute need, this work aims to face the grand challenge of providing wafer-scale photonic–atomic integrated circuits using a CMOS inspired process, without the need for cumbersome glass blowing process and without the need for distillation process. Specifically, we have demonstrated the bonding of a glass wafer to a SIN wafer with several photonic devices separated into four dies. Each dye consists of an Rb dispenser pill that is laser activated. Following fabrication, we have measured and characterized light–atom interactions in these wafer-scale fabricated devices at elevated temperatures and observed their operation over several weeks. These devices were shown to be successful in bridging miniaturization, manufacturability, and integration gap by paving the way for cost effective and mass production of atomic–photonic circuitry.

## 2 Fabrication

In this work, we present a wafer-scale atomic–photonic platform based on anodic bonding between a SIN photonic circuitry wafer and a BF33 glass wafer containing pre-etched vapor chambers. This CMOS-compatible approach enables batch fabrication of multiple atomic–photonic chips in a single process flow, with hermetic sealing, high thermal stability, and precise die-level repeatability. Our prior studies on ACWGs were based on a cumbersome fabrication process. In short, Rb vapor was introduced into the cell devices by attaching a Pyrex cylinder to the chip with thermally cured epoxy adhesive, followed by connecting the cylinder to a turbo-molecular vacuum system (Figure S1). The assembly was subjected to a 24-h bake-out, achieving a base pressure of 10^−7^ Torr. Natural Rb was then loaded into the cells (Figures S2 and S3), followed by isolation and hermetic sealing [13], [18], [28]. This fabrication approach presents several limitations: First, it is based on a manual processing. Each cell requires individual assembly, limiting scalability. Furthermore, a glass blowing process is needed for the distillation process. Another issue is the need to introduce a bulky and expensive Pyrex cell for each device. And another troubling issue is related to thermal constraints. The epoxy adhesive degrades above 110 °C, compromising vacuum integrity due to out-gassing and loss of sealing properties. Furthermore, the yield of this process is limited.

To overcome these limitations, we hereby describe a wafer-scale fabrication method that overcomes these disadvantages. Our approach is based on bonding on two wafers, the photonic integrated wafer and the vapor cell wafer. This is done by anodic bonding, also known as “field-assisted sealing” [29]. In its most common implementation, anodic bonding enables hermetic sealing between silicon and borosilicate glass (BF33). This technique has been successfully demonstrated for the fabrication of vapor cells with centimeter- and millimeter-scale dimensions [1], [12]. By adopting this approach, we overcome the thermal limitations imposed by epoxy-based sealing methods and enable reliable operation at elevated temperatures. Subsequently, we advance the technology toward CMOS-compatible, wafer-scale mass production – a capability that, to date, has only been demonstrated for millimeter-scale cells [21], [22]. This approach also addresses the challenges of high-cost and manual device fabrication.

**Figure 2:**
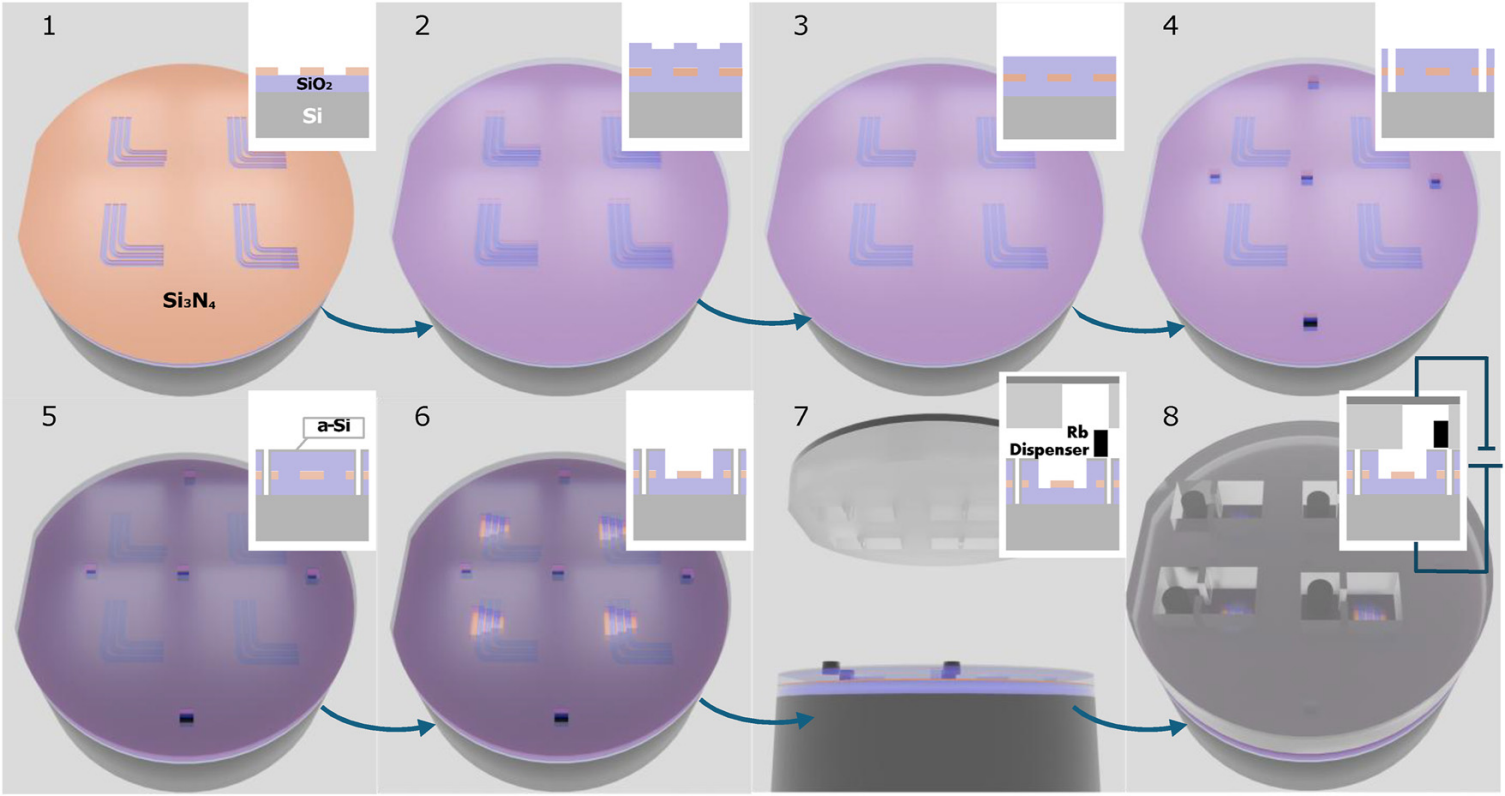
Fabrication process: (1) Fabricating waveguides by E-beam lithography and RIE of a 250-nm thick SiN layer on top of a 2-μm SiO_2_ layer on top of a 0.5-mm 4″ Si substrate wafer; (2) adding 2.5 μm PECVD SiO_2_ cladding; (3) 500 nm of CMP for planarization; (4) etching vias down to the Si substrate; (5) evaporating a 220-nm a-Si layer to short the surface with the Si substrate; (6) creating an interaction region for light with Rb by etching the SiO_2_ cladding above the waveguides; (7) prepare the second glass wafer made of two welded glasses, a 0.3-mm plane BF33 wafer, and 1.1 mm BF33 with drilled holes and microchannels for the Rb vapor; (8) anodic bonding for the Si and glass wafers with the Rb pill in the reservoir (AML) by applying a voltage of 800 V, a current limitation of 4 mA, and 500 N pressure at a temperature of 370 °C and vacuum of 10^−7^ mbar.

Our wafer-scale fabrication process is illustrated in Figure 2. We start with a 4-inch, 500-μm thick silicon wafer covered with a 2-μm thermal oxide and 250-nm low-pressure chemical vapor deposition grown Si_3_N_4_ layer. After cleaning in piranha solution (H_2_SO_4_:H_2_O_2_ 3:1), we spin-coated the wafer with ZEP520 and define the desired pattern, which consists of waveguides using an online lithography toolbox [30] and electron beam lithography (Elionix). The exposed regions were etched by reactive ion etching (RIE, Corial) (Figure 2-1). We have fabricated waveguides of different lengths, with the same cross-section of 700 nm width and a 250 nm height, supporting a single TE mode.

**Figure 3:**
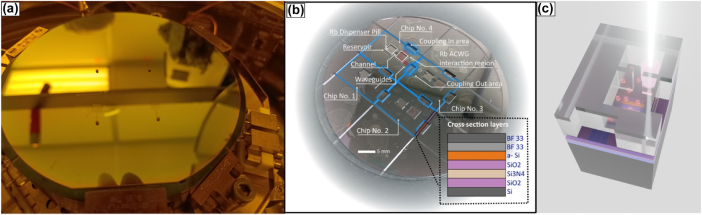
From wafer-level fabrication to chip-scale ACWG devices. (a) Photograph of the lithographically patterned silicon wafer, with an a-Si top layer, positioned inside the wafer bonder chamber prior to anodic bonding. Rb dispenser pills are aligned atop the designated reservoir sites. (b) Photograph of the bonded wafer after dicing showing four individual devices. Key features are annotated: reservoir (Rb dispenser pill), ACWG interaction region, microchannel, and optical coupling areas. Inset (not in scale): Cross-sectional schematic of layer stack (from bottom-up): Si substrate (500 μm), SiO_2_ (2 μm), SiN waveguide (250 nm), SiO_2_ (2 μm), a-Si (220 nm), and two layers of BF33 glass (1.4 mm). (c) Illustration of a diced photonic ACWG device during Rb dispenser pill activation.

**Figure 4:**
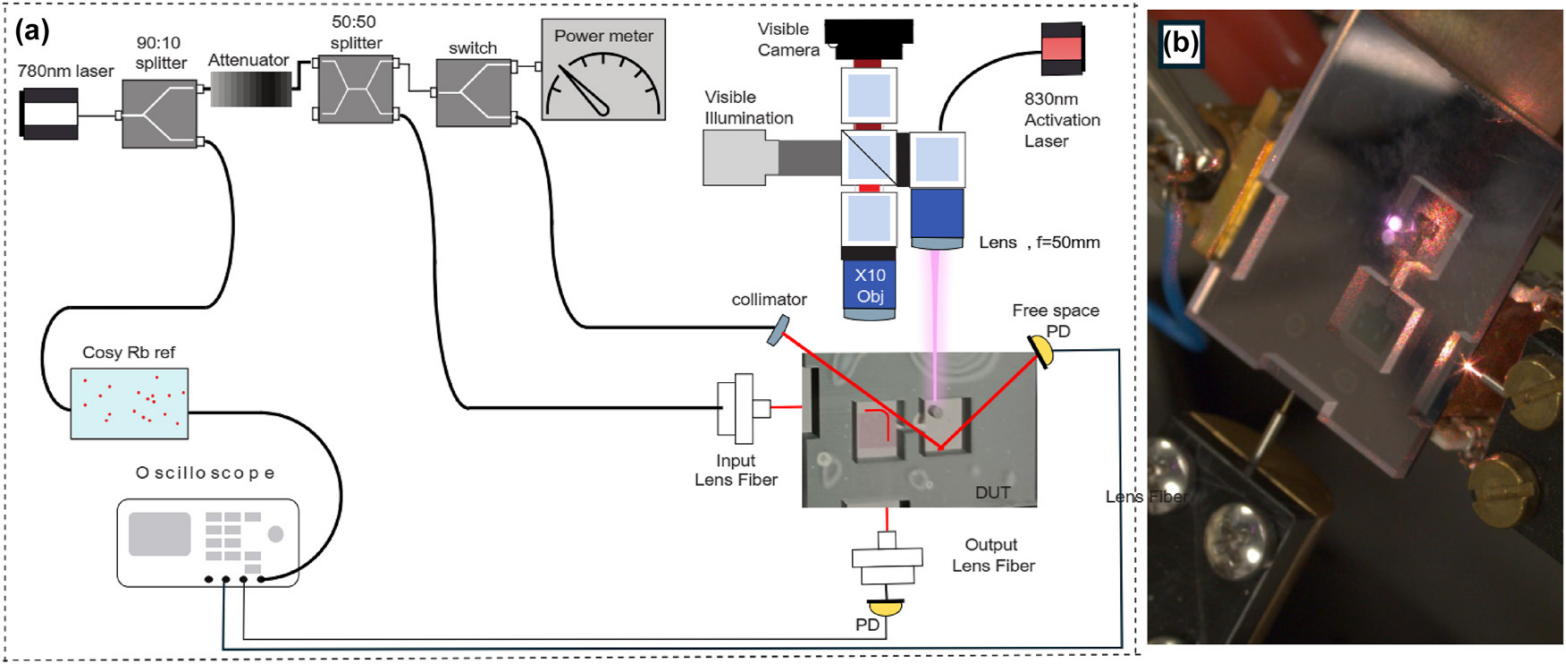
Measurement setup. (a) Schematic of the experimental setup for simultaneous Rb dispenser activation and measurement. The setup enables *in situ* saturated absorption spectroscopy during activation, as well as optical characterization via both free-space and waveguide-coupled configurations. (b) Photograph of the device under activation, showing the arrangement for concurrent free-space and waveguide measurements. The laser-heated pill is emitting bright red.

**Figure 5:**
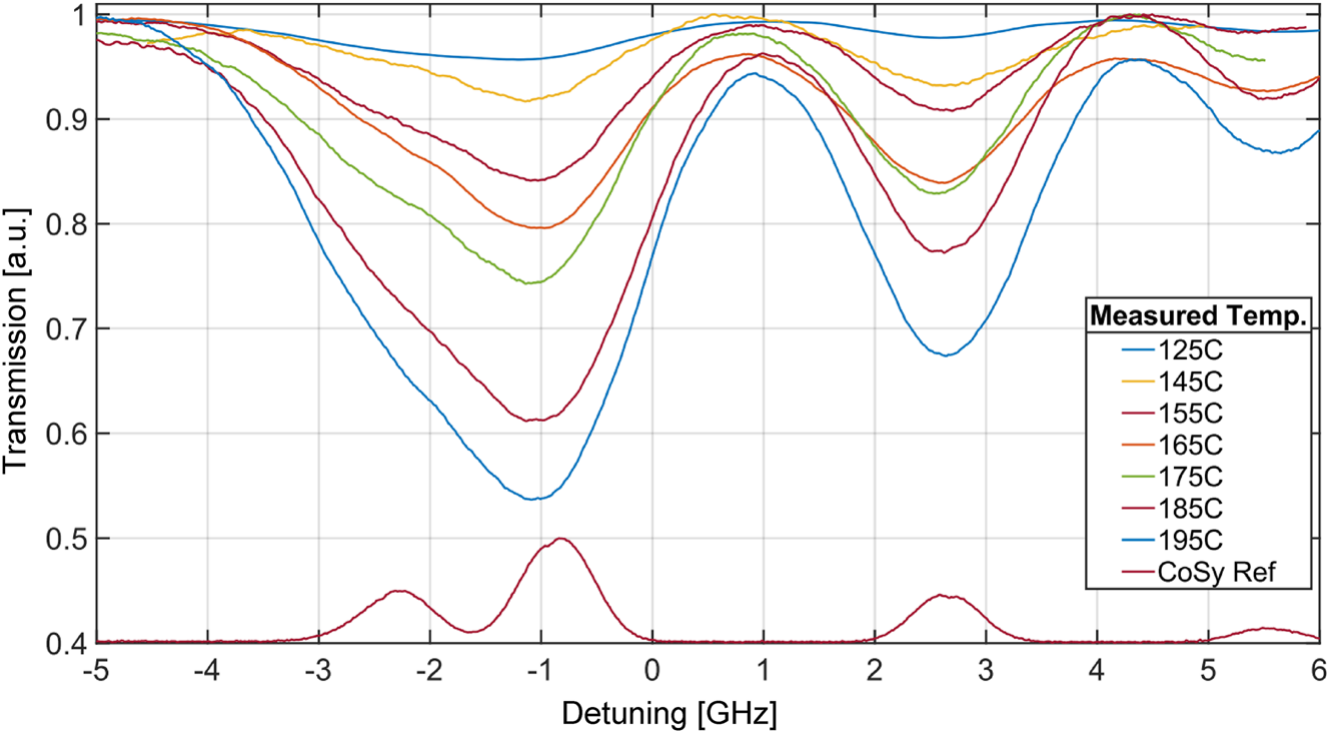
Evanescent absorption spectra measured in a 200-μm interaction region of the ACWG device with Rb vapor, using a constant input power of 50 μW at 780 nm. Measurements were performed from 125 °C to 195 °C in 10 °C increments. Increased absorption contrast observed at higher temperature, consistent with an enhanced atomic density. Resonance broadening increases by transit time and Doppler broadening effects.

**Figure 6:**
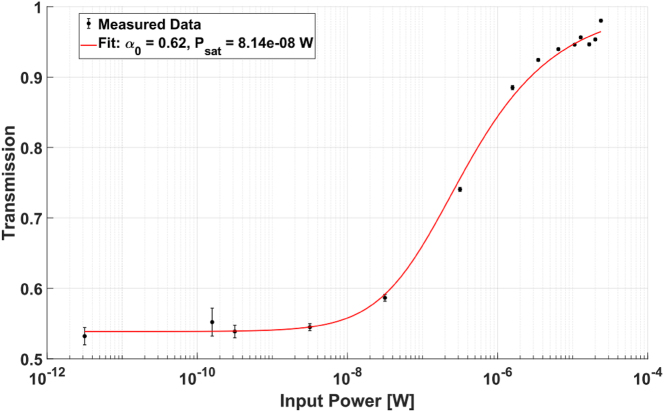
Minimal transimission of the ^85^Rb *F* = 2 → *F*′ = 2/3 transition as a function of the optical power within the ACWG for 200-μm interaction region at 185 °C. The estimated saturation power is 81 nW.

We then deposited a 2.5-μm-thick layer of silicon oxide on top using plasma-enhanced chemical vapor deposition (PECVD) (Figure 2-2). The waveguide pattern in the nitride layer was transferred to the upper oxide cladding, forming micro-trenches that can result in leaks inside the vacuum-sealed vapor reservoir. To assess this, we measured the SiO_2_ cladding surface profile above the waveguides using a mechanical profilometer (Veeco Dektak 150) and observed trench-like channels alongside each waveguide (Figure S4). To eliminate these channels, we deposited an additional 500 nm SiO_2_ cladding and planarized it by chemical-mechanical polishing (CMP). The thickness of each layer was measured before and after CMP (Mikropack, NanoCalc 2000), confirming the intended material removal and uniformity. Post-CMP profilometry verified that the trench structures vanished, and AFM measurements confirmed a surface roughness below 0.5 nm (Figure 2-3), suitable for reliable anodic bonding. After cleaning the wafer, we used a laser writer to define patterns in an AZ4562 resist for vias openings, followed by ICP-RIE of all three layers (PECVD oxide, nitride, and thermal oxide) down to the silicon substrate (Figure 2-4). Then, we deposited about 220 nm PECVD amorphous silicon (a-Si) (Figure 2-5) to create an electrical contact between the conductive silicon substrate and the top surface (the amorphous silicon) for the anodic bonding [31]. The use of a deposited layer such as amorphous silicon as a binder allow us to bond an oxide cladded wafer to glass and can potentially support many other wafer technologies such as lithium niobite on the insulator. The area of Rb vapor and ACWG interaction was then defined by a laser writer, followed by an oxide wet etching by a buffered HF solution (BOE) (Figure 2-6). At this point, the first wafer for the anodic bonding is ready.

The second glass wafer must satisfy several requirements: First, it must be transparent at the activation illumination wavelength to generate sufficient heat corresponding to the operational requirements of a commercially available alkali dispenser (SAES Rb/AMAX/Pill1-0.6). Second, the wafer must have sufficient thickness to allow for the fabrication of a reservoir capable of accommodating the Rb dispenser (Rb dispenser dimensions are 1 × 0.6 mm). Third, the thermal expansion should be well matched to the silicon thermal expansion. Finally, the contact surface should be polished to a minimal roughness to facilitate anodic bonding sealing.

Indeed, there are several established approaches for fabricating reservoir cavities in glass, each with distinct limitations that we evaluated experimentally. Mechanical drilling, for example, often results in poor surface quality and can introduce cracks due to the deformation of glass under the thrust force of the drill, particularly at the exit surface [32]. Additionally, this method does not meet the transparency requirements for our application. Water jet micromachining was also tested; however, the resulting holes exhibited highly irregular surfaces, non-uniform inner walls, and residual particles adhering to the glass, most likely due to the high operating pressures involved. These surface imperfections complicate subsequent bonding steps [12]. Wet etching, typically using hydrofluoric acid (HF), is another option, but for 1-mm thick glass, the process is very slow – typical etch rates range from 0.07 μm/s to 0.2 μm/s, requiring up to 4 h for complete etching. Furthermore, the isotropic nature of wet etching in amorphous glass leads to rounded sidewalls and low aspect ratios, and the process is prone to defects such as pinholes and notching, primarily due to residual stress in the masking materials [33]. Deep reactive ion etching (DRIE) was also considered, but it exhibits a low etch rate making it impractical for fabricating millimeter-scale features, as etching a 1 mm-thick glass would require many hours.

Given those limitations, we adopted a hybrid approach: bonding two separate Borofloat 33 (BF33) glass wafers [34] of different thicknesses. The first, a 1.1-mm-thick wafer, was used to fabricate through-holes for the dispenser reservoirs, while the second, a 0.3-mm-thick wafer, served as a transparent window.

**Figure 7:**
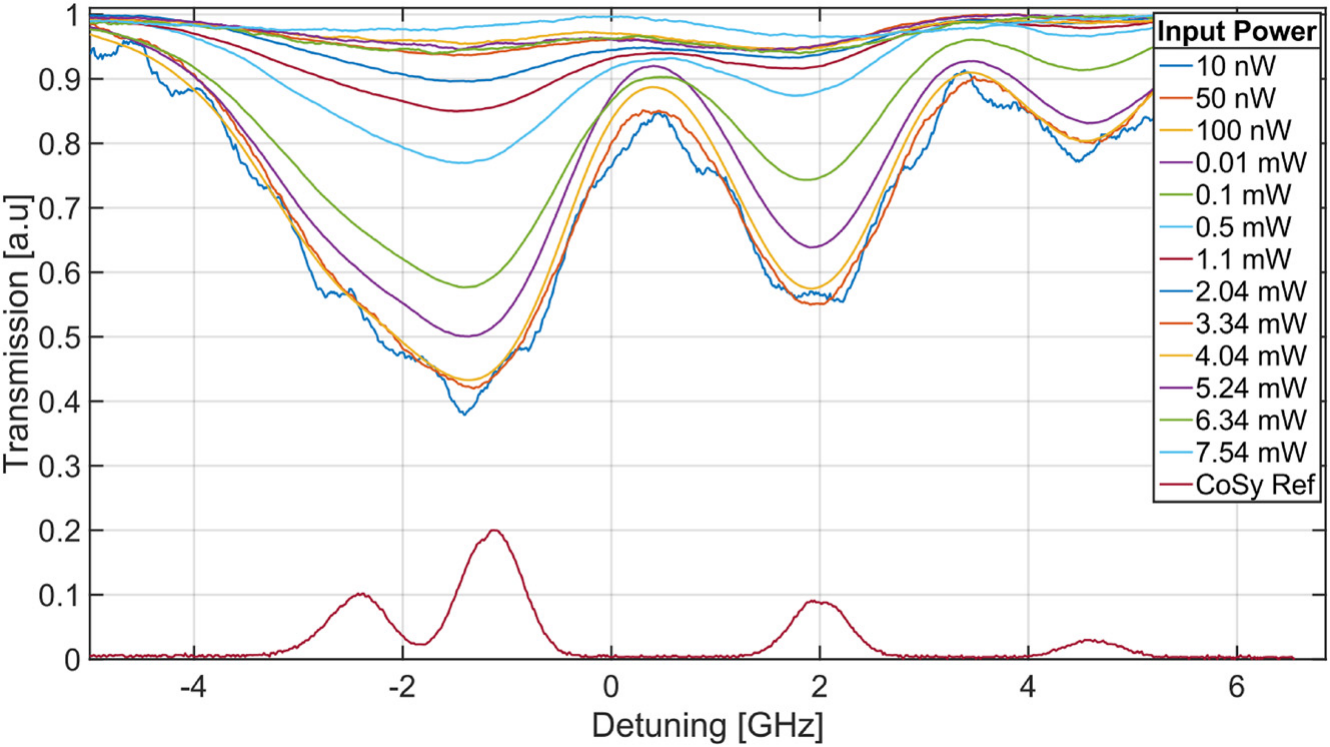
Measurement of devices of the 200-μm interaction region of Rb at 185 °C.

Glass-to-glass bonding can be achieved using several techniques, including thermocompression [35] or plasma-assisted hydrophilic glass-to-glass direct bonding [36], [37]. However, to minimize the risk of contamination from bonding agents or residual molecules that could interact with the Rb vapor, we elected to bond the two BF33 glass wafers using laser welding technology (Figure 2-7). This approach enables a robust, hermetic seal without introducing additional materials into the device environment.

Subsequently, anodic bonding was carried out using a wafer bonder (AML AWB-04). Prior to bonding, both wafer surfaces were thoroughly cleaned with piranha solution, and the Rb dispenser was carefully positioned within the reservoir of each device. The wafers were then loaded into the bonding chamber, which was evacuated to a base pressure of 10^−7^ Torr and simultaneously baked at 370 °C for 24 h to remove residual moisture. The anodic bonding process was conducted at 370 °C, with an applied voltage of 800 V, a current limit of 4 mA, and an applied force of 500 N (Figure 2-8). Figure 3-a shows the lithographically patterned silicon wafer, with an a-Si top layer, positioned inside the wafer bonder chamber prior to anodic bonding. Rb dispensers are aligned atop the designated reservoir sites.

Alignment marks patterned at identical positions on both the glass and silicon wafers enabled accurate alignment (∼+/−2.5 μm accuracy) prior to the bonding step. Figure 3-b presents the post-dicing bonded wafer containing four individual devices fabricated from the 4-inch substrate. The image delineates the following functional regions within each device: reservoir housing the Rb dispenser pill, ACWG interaction region where atom–light interactions occur, a microchannel connecting the reservoir to the waveguide region (microchannel dimensions – width: 500 μm, length: 2,400 μm, height: 1.1 mm), optical coupling interfaces consisting of input/output grating couplers for light injection and collection. Inset describing (not in scale) cross-sectional schematic of the layered structure (from bottom-up): Si substrate (500 μm), SiO_2_ (2 μm), SiN waveguide (250 nm), SiO_2_ (2 μm), a-Si (220 nm), and two welded layers of BF33 (1.4 mm). We performed several wafer-bonding runs and obtained repeatable results with no evident cracks or stitching-related defects.

A fiber-coupled diode laser (830 nm, K830F02FN-2.000W) was used to activate the Rb dispenser. The beam was focused onto the pill surface with a 50-mm focal length lens. Figure 3-c illustrates a photonic, diced, ACWG device during Rb dispenser pill activation. During activation, the diode laser was driven at 1 A, delivering 0.38 mW of optical power for 30 s. The chip was maintained at 120 °C throughout the process to prevent condensation of atoms on the waveguides.

## 3 Atomic clad waveguides (ACWGs) – experimental characterization

After device fabrication and Rb dispenser activation, we characterize the optical and atomic properties of the integrated chip devices. The experimental setup was designed to simultaneously enable precise alignment of optical lens fibers to the chip facets, temperature control, and monitoring of spectroscopic optical signals. In the following subsections, we detail the measurement configurations, procedures, and data analysis methods used to evaluate device performance. A narrow-linewidth 780 nm laser (Toptica) was fiber-coupled to a 90:10 splitter. The low-power output was directed to a reference Rb cell for reference and calibration, while the higher-power arm was sent through an optical attenuator and then to a 50:50 splitter. One output of this splitter was connected to an optical switch, which routed the light either to a power meter or to a fiber collimator for free-space spectroscopy measurements. During Rb dispenser activation, the output from the chip’s reservoir area was reflected onto a photodetector (PD) equipped with a 780-nm bandpass filter. This configuration enabled *in situ* saturated absorption free space spectroscopy, allowing us to monitor the Rb vapor density and try to avoid overactivation of the dispenser [12]. Figure S5 shows the initial Rb free space absorption signal recorded immediately after pill activation. Following the detection of free-space Rb spectroscopy via reflected light from the chip, the switch was set to direct the output to a power meter. The second output of the 50:50 splitter was fiber-coupled to the input facet of the device, injecting light into the waveguide via edge coupling to an inverse taper. The guided light then interacted with the Rb atoms in the interaction region through the evanescent field and was subsequently collected at the output facet by another inverse taper and coupled via lensed fiber to a photodetector. Since the start of the activation process, the device under test was placed on top of an oven to maintain the desired temperature, which was applied to both the reservoir and the interaction region. In Figure 4-a, we present a schematic of the simultaneous Rb dispenser activation and measurement setup. Figure 4-b shows a photograph of the device during activation, illustrating the configuration for concurrent free-space and waveguide measurements.

The fabricated device contains several SIN waveguides. The waveguides have a different length of exposure to the Rb vapor, which is predefined in the SiO_2_ etching mask step of the fabrication (Figure 2-6). The overall coupling loss, measured at 170 °C, was found to be ∼50 dB, including coupling loss from the fiber to the waveguide (two facets) and from the oxide-clad waveguide to the ACWG (two facets). We attribute the high losses also to the condensation of Rb atoms or other materials ejected from the Rb pill during activation. Other possible reasons for the high loss are the instability of the edge coupling measurement at high temperature due to thermal expansion and hot air flow, as well as low dicing quality of the facets. The loss mechanisms are now being studied, with the goal of finding the exact loss mechanisms and achieving significant loss reduction in future devices. We have fabricated waveguide with interaction lengths varying from 200 μm to several millimeters but were only able to observe reasonable transmission for devices with interaction lengths of 200 and 500 μm. After the activation, we kept the device heated to 100 °C permanently to generate a temperature gradient between the Si chip and the glass cover and prevent any further condensation of Rb on the surface [38]. In Figure 5, we present measurements of evanescent absorption in the ACWG device with a 200 μm interaction length using a constant input power of 0.2 mW at 780 nm. The measurements were performed at various temperatures, ranging from 125 °C to 195 °C in 10 °C increments, under conditions where the optical transitions are not saturated. As expected, the absorption contrast increases with the temperature. Additionally, when compared to the reference signal from the Rb cell (Cosy), the broadening of the Rb resonance becomes more pronounced at higher temperatures. This broadening can be attributed to both the finite transit and the Doppler effect, with Doppler broadening being the dominant mechanism in this case [13], [14], [39].

We repeated the measurements for a longer ACWG interaction length of 500 μm, as shown in Figure S6. Like the results obtained with the shorter interaction length, we observed consistent behavior both in the saturation region and at maximum absorption. Notably, the longer interaction length resulted in improved absorption contrast at lower input power, which can be attributed to the increased interaction between the evanescent guided light and the Rb atoms along the extended ACWG region.

Next, we investigated the nonlinear interaction in the ACWG by measuring the absorption spectrum as a function of incident light intensity, which was controlled using an attenuator placed before the 50:50 splitter. In Figure 6, we present the transmission spectra obtained for a 200-μm interaction length at a temperature of 185 °C. Under these conditions, we observed a high absorption contrast at a low input optical power (10 nW) and clear saturation behavior at higher input power.

To estimate the saturation power, we analyzed the contrast data for the ^85^Rb *F* = 2 → *F*′ = 2/3 transition at 185 °C as a function of input power. The data were fitted to the exponential function: $T\left(P\right)=\exp\left(-\alpha_{0}{\left(1+\frac{P}{P_{\text{sat}}}\right)}^{-0.5}\right)$ , where *α*_0_ is the optical density corresponding to the unsaturated level measured, *P* is the optical power level within the ACWG at resonance (obtained by a linear fit around the resonance), and *P*_sat_ is the saturation optical power [13]. The fit, shown in (Figure 7), yields an estimated onset of saturation of 81 nW.

We repeat these ^85^Rb *F* = 2 transition measurements for different temperatures. In Figure S7, we present a series of measurements for a 200-μm long waveguide showing the effects of varying both temperature and input power. Similarly, Figure S8 displays corresponding measurements for a 500-μm interaction length.

## 4 Discussion

In summary, we have demonstrated a new platform of wafer atomic photonic integrated circuits that are integrated with a wafer of hot vapor cells filled with Rb pills as the vapor source. We have used this platform to fabricate and demonstrate wafer-scale ACWGs operating at unprecedently high temperatures of up to 195 °C.

Temperature- and power-dependent spectroscopic measurements were used to characterize atom–light interactions, revealing Doppler broadening as the dominant spectral broadening mechanism. Importantly, no significant spectral broadening attributable to contamination or residual byproducts from the anodic bonding process or the Rb dispenser was observed. The device was measured over several weeks of nearly continuous operation without showing noticeable degradation.

Waveguide losses are currently noticeable and are attributed to the condensation of Rb vapor on the waveguide. The issue of condensation is known [38], [40] and seems to be more severe in microfabricated cells. We intend to take several steps to tackle this effect, including the use of light-induced atomic desorption [40], [41], [42] and alumina deposition [43]. A complementary solution will be to minimize the amount of Rb being sputtered at the moment of activation, e.g., by precisely control the optical power required for activation, blocking any direct line of sight between the pill and the photonic devices, and studying different Rb placement technics [21], [44] where devices are isolated at the moment of activation.

To further reduce the overall optical loss, future device iterations will focus on improving facet quality by enhancing facet smoothness through optimized dicing and polishing procedures, or alternatively by implementing a deep RIE facet-etch process to define clean, vertical facets. In addition, replacing the current oxide wet-etch with an ICP-PECVD oxide lift-off approach is expected to improve the quality of the interface between the region with top oxide cladding to the region where the top oxide is removed, thereby reducing scattering losses. It may also be that the silicon–glass anodic bonding at 370 °C produces mechanical stress in the waveguides, leading to loss and perhaps also refractive index modulation and birefringence. This will be studied in more detail in future devices.

While challenges remain, the demonstrated platform holds an enormous potential to become an enabling technology in wafer-scale integrated atomic devices, allowing miniaturization, high level of integration, manufacturability, and low-cost devices. This will pave the way for the use of such devices in applications ranging from frequency referencing, wavelength meters, laser stabilization, and atomic clocks to magnetic and electric field sensing.

## Supplementary Material

Supplementary Material Details
