# Supplementary material for: Wafer-scale integration of photonic integrated circuits and atomic vapor cells
Source: Nanophotonics. 2025 Dec 5;14(27):5545–54. doi: 10.1515/nanoph-2025-0500 (PMC12717930; doi:10.1515/nanoph-2025-0500)
Supplement: Supplementary file 1 — Supplementary Material Details [file j_nanoph-2025-0500_suppl_001.pdf]

Supplementary material for Wafer-scale integration of photonic integrated circuits and atomic vapor cells

Arieh Grosman[1], Roy Zektzer[2], Noa Mazurski[1], Liron Stern[1], and Uriel Levy[1*]
1 Institute of Applied Physics, The faculty of science, The Center for Nanoscience and Nanotechnology, The Hebrew University of Jerusalem, Jerusalem, Israel
2 Faculty of Engineering, Bar-Ilan University, Ramat-Gan 5290002, Israel
Email: Arieh.grosman@mail.huji.ac.il
*Corresponding author: ulevy@mail.huji.ac.il

This supplementary material contains information on device fabrication and device installation within the experimental setup.

S1. Previous generation of distilled ACWG device fabrication

In this section, we describe in detail the previous generation of the distilled atomic-cladding waveguide (ACWG) device fabrication process used in our lab. After the lithography and dicing steps of a single chip, the procedure for introducing Rubidium (Rb) vapor into the cell devices was carried out as follows. A Pyrex cylinder was glued to the chip using a thermally cured epoxy. The cylinder contained natural Rb powder, and its opposite end was connected to a turbo-molecular vacuum system (Fig. S1). The entire assembly was baked at 500 °C for 24 h, after which a base pressure of 10^{-7} Torr was achieved. At this stage, the Rb was distilled into the cells (Fig. S2–S3), and the cells were subsequently isolated by disconnecting them from the Pyrex cylinder.

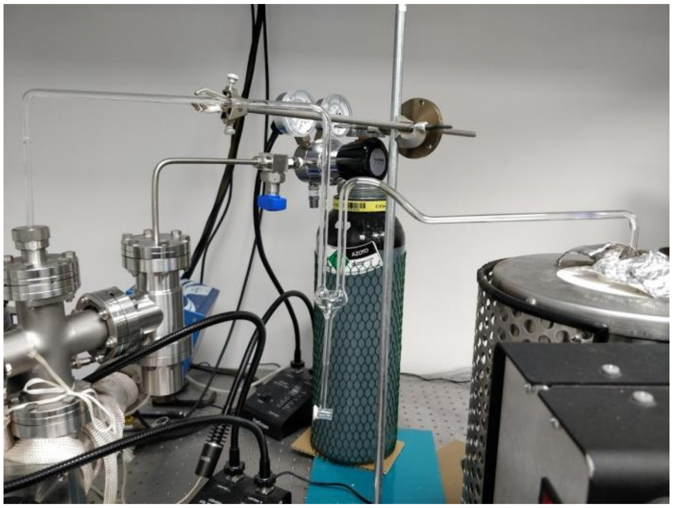

Fig. S1**: fabrication setup for distilled ACWG.** photograph of The Pyrex cylinder with natural Rb powder which were placed inside a 500°C oven, and the overside was connected to a turbo vacuum system, with glued cell using epoxy

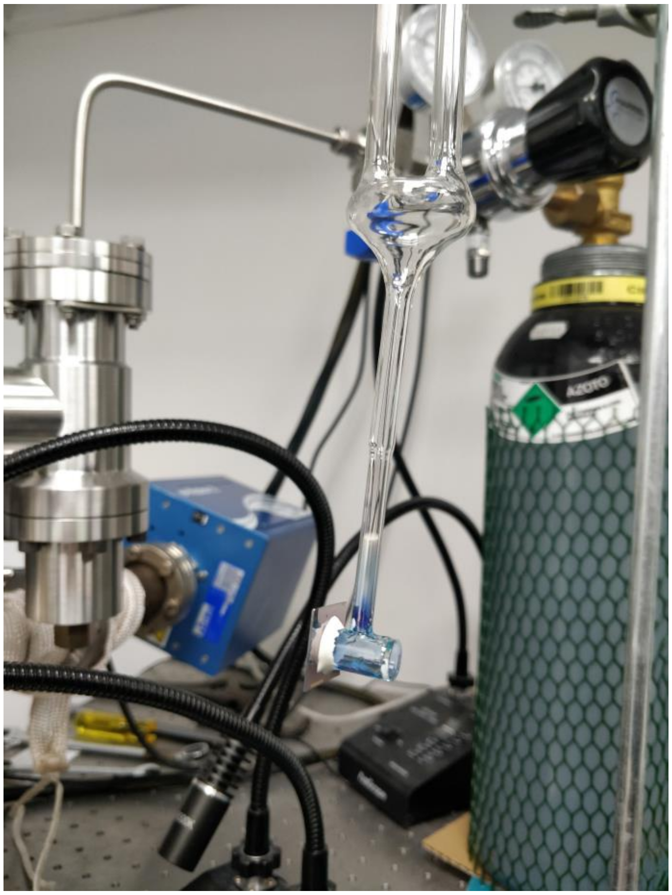

Fig. S2: photograph of the glued cell after lunching Rb vapor inside the cell

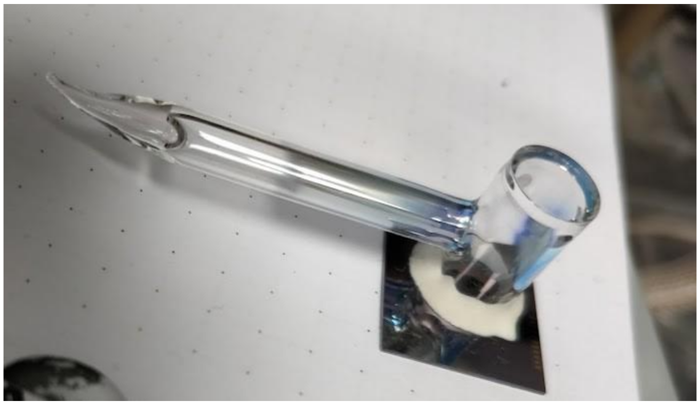

Fig. S3: photograph of the glued ACWG cell after disconnection from the cylindric Pyrex with Rb vapor inside the photonic cell

S2. Wafer-scale ACWG device fabrication

To avoid potential air leaks inside the vacuum-sealed vapor reservoir, we characterized the SiO_2 cladding surface above the waveguides using a mechanical profilometer (Veeco Dektak 150). The measurements revealed trench-like features beside each waveguide, with typical dimensions of approximately 4 μm in width and 170 nm in height (Fig. S4). These micro-trenches originate from pattern transfer from the nitride layer into the oxide and serve as air-leak channels during bonding, as we verified through leak tests. To eliminate this issue, we deposited an additional 500 μm SiO_2 cladding and planarized the surface by chemical–mechanical polishing (CMP). The thickness of each layer was measured before and after CMP (Mikropack NanoCalc 2000), confirming the intended material removal and uniformity. Post-CMP profilometry verified that the trench structures were completely removed, and AFM measurements showed a surface roughness below 0.5 nm (Fig. 2–3), ensuring suitability for reliable anodic bonding.

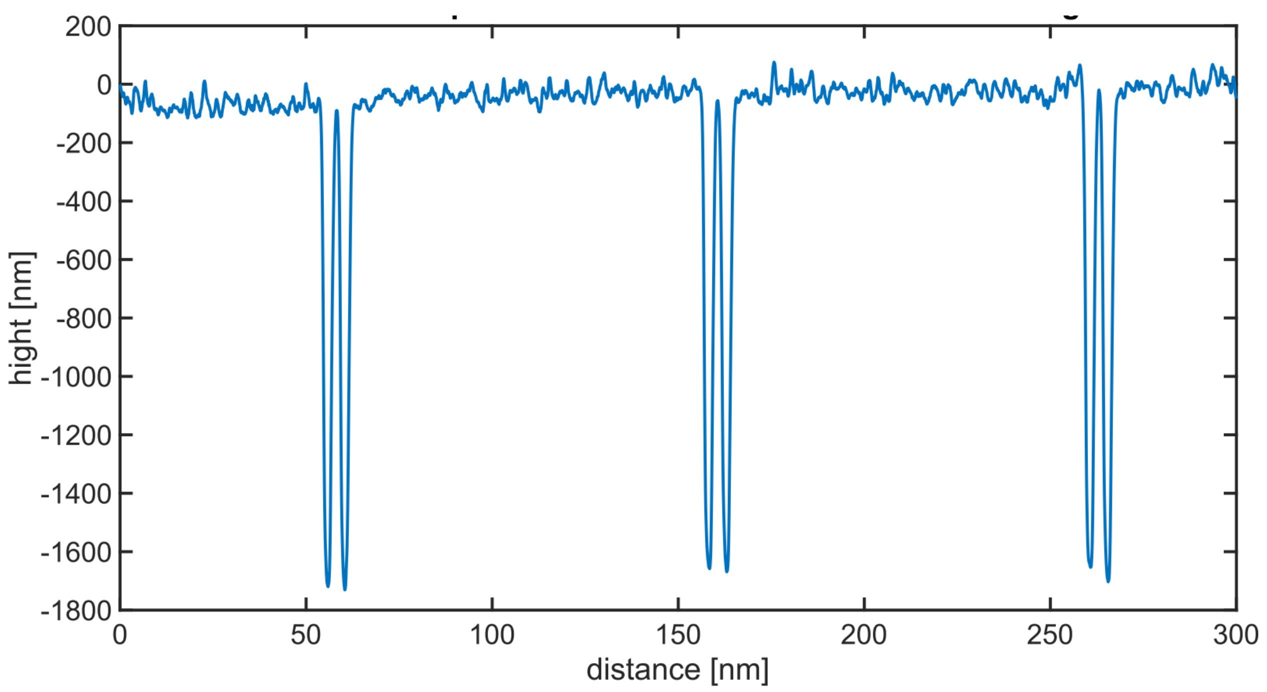

Fig. S4: Mechanical profilometer measurement of the SiO_2 surface above the waveguides, revealing trench-like features that can serve as air-leak channels.

S3. Anodically bonded ACWG chip additional measurement

To further characterize the performance of the anodically bonded ACWG devices, we conducted a series of free-space and on-chip spectroscopy measurements across different device lengths, temperatures, and optical input powers. Figure S5 shows the initial free-space reflection Rb spectroscopy collected during pill activation, confirming successful Rb release and the onset of vapor absorption features. Figures S6–S8 present on-chip transmission measurements for devices with 200-μm and 500-μm interaction regions under a range of operating temperatures. Fig. S6 shows the 500-μm devices measurement at temperature of 185 °C, in different injection power to the ACWG and we observed consistent behavior in both the saturation region and at maximum absorption. In addition, the 200-μm and 500-μm devices were characterized from 125 °C to 200 °C under varying optical input powers for ^{85}RbF=2 transition (Figs. S7–S8). The power–dependent spectra, plotted on a logarithmic scale, reveal in low-injection-power regime, clear temperature-induced changes in absorption depth, consistent with increasing atomic density.
However, we observe that in the high-injection-power regime—where the Rb transitions approach saturation and the resonant absorption become less sensitive to input power—the overall optical loss exhibits only minor variation for the 200-μm devices. In contrast, the 500-μm devices show more pronounced variations in transmission. This behavior suggests stronger sensitivity to Rb condensation or accumulation along longer interaction lengths.

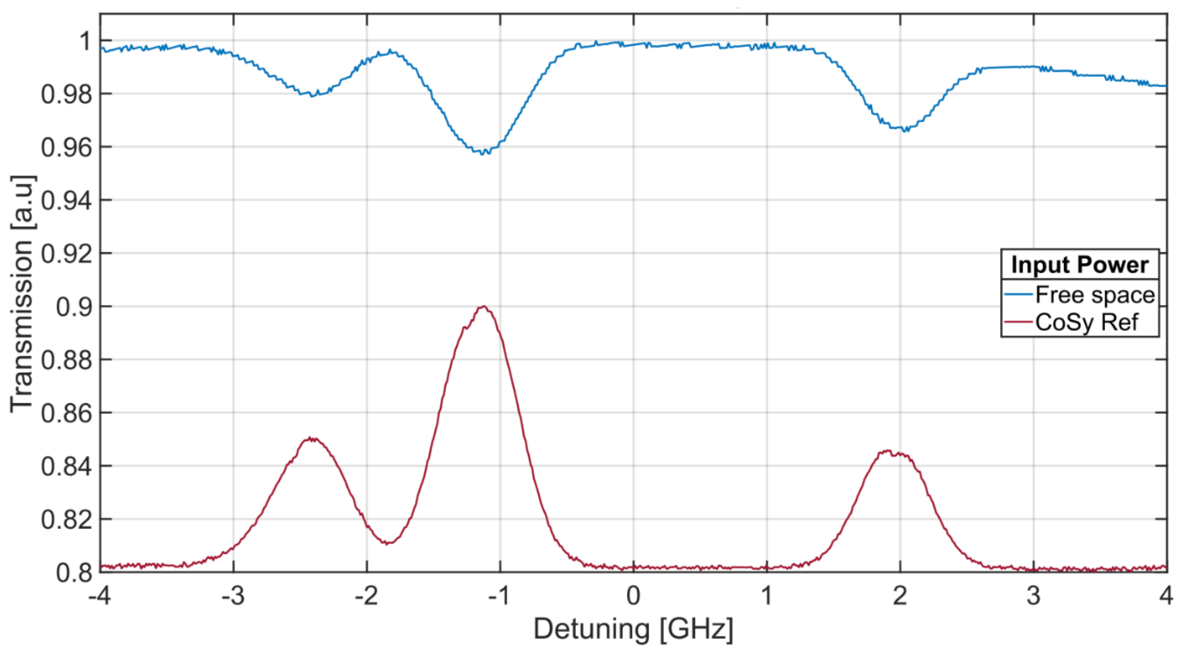

Fig. S5: Initial free-space reflection Rb spectroscopy during pill activation.

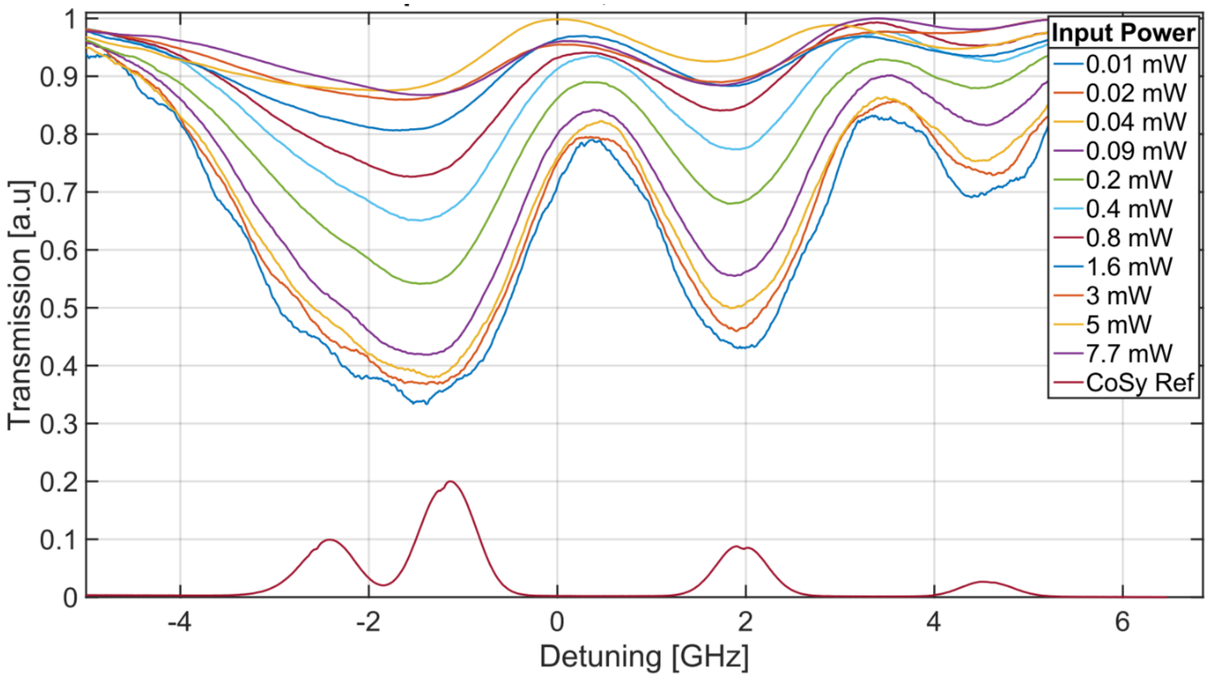

Fig. S6: Measurement of devices with a 500-μm interaction region at 185 °C.

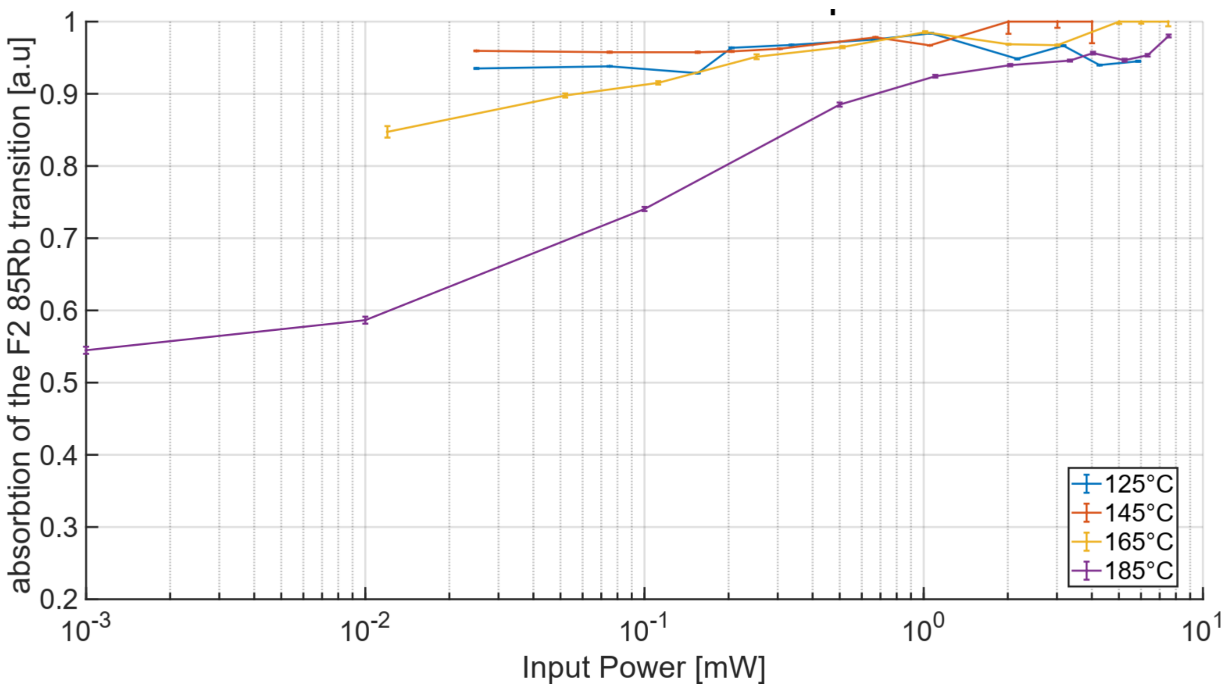

Fig. S7: Measurement of devices with a 200-μm interaction region at 125 °C, 145 °C, 165 °C, and 185 °C under different input powers on a logarithmic scale.

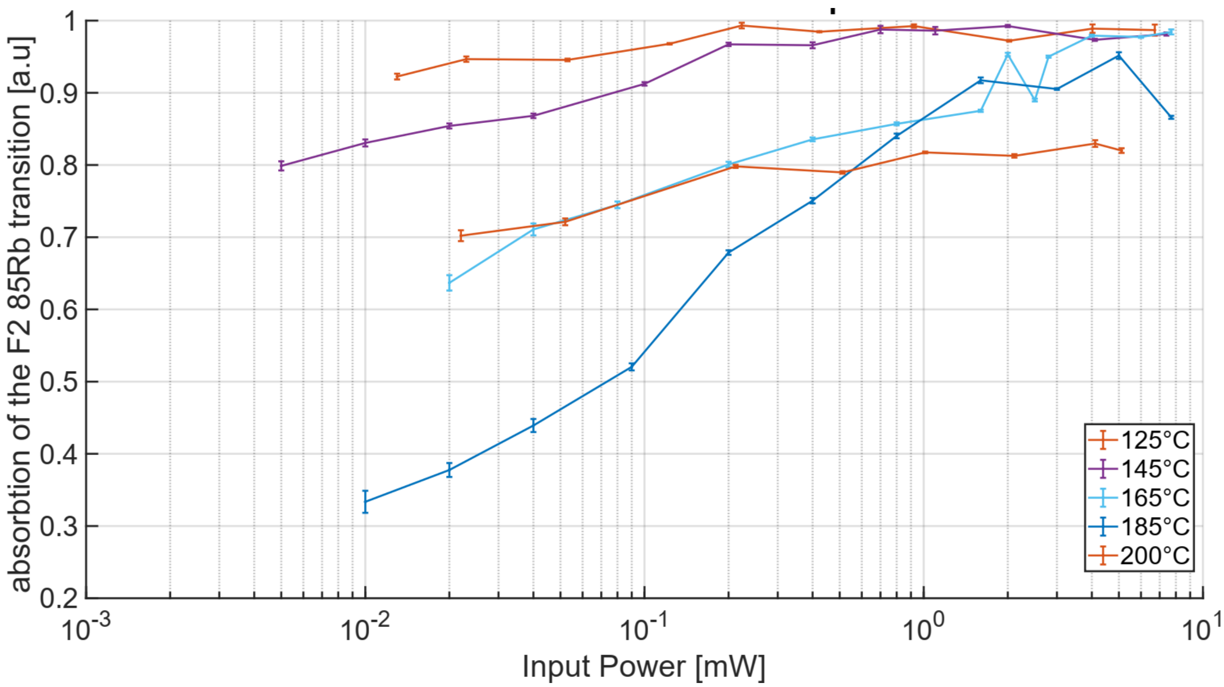

Fig. S8: Measurement of devices with a 500-μm interaction region at 125 °C, 145 °C, 165 °C, 185 °C, and 200 °C under different input powers on a logarithmic scale.

S4. Comparison between previous and current ACWG fabrication technology

Fabrication approach	**4" Wafer level anodic bonding**	**Chip scale gluing**
Number of devices per fabrication process	4-10	1
Device size	<5x5x2mm	20x20x20 mm
Operation temperature	>200C	<110C

Table I: Comparison of current and previous fabrication method of ACWG , for number of fabricated devices in single batch, Device size, and operational temperature.
